# The effects of oral and vaporized cannabis alone, and in combination with alcohol, on driving performance using the STISIM driving simulator: A two-part, double-blind, double-dummy, placebo-controlled, randomized crossover clinical laboratory protocol

**DOI:** 10.3389/fphar.2022.964749

**Published:** 2022-09-06

**Authors:** C. Austin Zamarripa, Matthew D. Novak, Elise M. Weerts, Ryan Vandrey, Tory R. Spindle

**Affiliations:** Behavioral Pharmacology Research Unit, Johns Hopkins University School of Medicine, Baltimore, MD, United States

**Keywords:** cannabis, alcohol, driving simulation, field sobriety tests, vaporized cannabis, oral cannabis

## Abstract

The legalization of cannabis for medicinal and non-medicinal purposes, and the corresponding increase in diversity of cannabis products, has resulted an urgent need for cannabis regulatory science. Among the most pressing needs is research related to impairment due to cannabis exposure, especially on driving performance. The present project was designed to evaluate the impact of oral and vaporized cannabis, when administered alone or in combination with alcohol, on simulated driving performance (STISIM driving simulator), cognitive/psychomotor ability, and field sobriety performance. Healthy adults will complete two, double-blind, double-dummy, placebo-controlled, randomized crossover clinical laboratory studies, one with oral cannabis (16 men/16 women) and the second with vaporized cannabis (16 men/16 women). In each study, participants will complete seven experimental sessions during which acute doses of placebo or high Δ9-THC cannabis containing 0, 10, or 25 mg Δ9-THC will be administered both alone and in combination with placebo or alcohol-containing beverages (target breath alcohol concentrations, BAC, of 0.0% or 0.05%). A positive control session (i.e., alcohol at target BAC of 0.08% with placebo cannabis) will also be completed. Simulated driving performance tests (available for download; see Methods), field sobriety assessments, subjective drug effect questionnaires, a mobile device impairment test (DRUID app), and collection of whole blood specimens will be completed repeatedly during each session. Linear mixed models will be used to test for differences across experimental conditions and *a priori* planned comparisons will be used to determine differences between conditions of interest (e.g., cannabis alone vs cannabis with alcohol). This research is designed to extend prior studies of cannabis and alcohol on driving performance by using oral and vaporized routes of cannabis administration. By increasing understanding of impairment associated with co-use of alcohol and these novel forms of cannabis, this research could inform impairment detection standards for cannabis and alcohol and have important implications for law enforcement, public policy decisions regarding accessibility of these substances, and education of the general population who may use cannabis and/or alcohol. Lastly, this manuscript provides interested researchers with access to the simulated driving scenarios and data extraction tools developed for this study as a means of facilitating future cross-study comparisons, which is important given the heterogeneity in methods used across laboratories in prior research.

## 1 Introduction

Cannabis (marijuana) and alcohol are two of the most used drugs of abuse in the world. In U.S. surveys conducted in 2019, approximately 48 million adults reported use of cannabis (Substance Abuse and Mental Health Services Administration, SAMHSA, 2021), and alcohol was used by approximately 140 million U.S. adults (SAMHSA, 2021). Alcohol use has a severe toll on public health; 28% of all fatal automobile accidents involve culpable drivers determined to have been under the influence of alcohol at the time of crash (National Highway Traffic Safety Administration, NHTSA, 2018). Like alcohol, cannabis that contains delta-9-tetrahydrocannabinol (Δ9-THC) can impair driving performance by disrupting psychomotor skills, cognitive abilities, and attention (Sewell et al., 2009; Spindle et al., 2018; Arkell et al., 2020), and detection of Δ9-THC concentrations in blood indicative of recent cannabis use is associated with a significant increase in crash risk (Preuss et al., 2021).

Coincident with increased cannabis legalization for medicinal and/or non-medicinal (“recreational”) purposes, there has been an increase in the incidence of driving under the influence of cannabis, (DUIC; Kelley-Baker et al., 2017). Increases in DUIC are likely due to decreased harm perceptions associated with cannabis use (Berg et al., 2015) as well as increased access to high Δ9-THC cannabis, which is steadily increasing in potency each year (Chandra et al., 2019). Of particular concern, an increasing number of people report regularly using alcohol and cannabis concurrently (i.e., “co-use”), most often in states where cannabis is legal (Kim et al., 2020), and many of these individuals report driving following alcohol-cannabis co-use (Fink et al., 2020). Importantly, when administered together, alcohol and cannabis produce additive or synergistic impairment (Sewell et al., 2009; Hartman et al., 2015; Yurasek et al., 2017), which is likely driven, in part, by pharmacokinetic interactions between alcohol and Δ9-THC (Hartman et al., 2016b). Individuals who have ingested both alcohol and cannabis also have a greater probability of being involved in a car accident and are more likely to be culpable for the accident than individuals who have ingested only alcohol or cannabis (Drummer et al., 2020).

Oral cannabis products (or “edibles”) and cannabis vaporizers have increased in popularity as cannabis legalization has expanded (Steigerwald et al., 2018; Schauer et al., 2020). However, the few studies that have administered alcohol and cannabis concurrently have almost all used a smoked route of administration for cannabis. When administered in the absence of alcohol, the time course and/or magnitude of the acute impairing effects of oral and vaporized cannabis differ markedly from smoked cannabis (for review, see Zamarripa et al., 2022). For example, peak subjective drug effects and impairment after ingestion of high Δ9-THC cannabis edibles occurs much later, and persists for longer (Spindle et al., 2021) than smoked high Δ9-THC cannabis (Wachtel et al., 2002). The pharmacokinetics of oral cannabis also differ from inhaled methods. For example, relative to inhaled cannabis, oral cannabis produces lower peak blood concentrations of Δ9-THC and higher concentrations of 11-OH-Δ9-THC (the primary psychoactive metabolite of Δ9-THC; Vandrey et al., 2017) as a result of first-pass metabolism (Huestis 2007). Despite key differences in the onset of effects and pharmacokinetics between oral and smoked high Δ9-THC cannabis, the peak magnitude of impairment between these two routes of administration is similar among occasional cannabis users (Newmeyer et al., 2017; Spindle et al., 2018; Schlienz et al., 2020). The acute effects of vaporized high Δ9-THC cannabis follow the same general time course as smoked high Δ9-THC cannabis (Abrams et al., 2007; Newmeyer et al., 2017; Spindle et al., 2018). However, the magnitude of impairment for vaporized cannabis is typically higher than smoked at a given Δ9-THC dose when dose delivery is complete for both routes of administration (Spindle et al., 2018). Taken together, differences in cannabis pharmacokinetics and profiles of impairment across routes of cannabis administration suggests oral and vaporized cannabis products may interact with alcohol in distinct ways from smoked cannabis and highlights the need for further co-use research.

The planned studies described here will extend prior alcohol/cannabis co-use studies by characterizing impairment associated with the co-use of alcohol and cannabis consumed orally or *via* vaporization. To date, no studies have characterized the effects of co-using alcohol and cannabis edibles, and only one study examined co-use of alcohol and vaporized cannabis. In the lone published study that evaluated the combined effects of alcohol (blood alcohol concentration, BAC, of 0.065%) and vaporized cannabis (14.5 mg and 33.5 mg Δ9-THC), the two drugs displayed additive effects on driving impairment; the increased impairment under co-use conditions was seemingly mediated by pharmacokinetic interactions between alcohol and cannabis (i.e., increased plasma Δ9-THC and 11-OH-Δ9-THC concentrations under co-use vs. cannabis-only conditions; Hartman et al., 2015). The present research will build on prior co-use studies with smoked cannabis to advance knowledge by dosing participants with alcohol doses targeting BACs that define legal intoxication in Europe (0.05% both alone and with cannabis) and most U.S. states (0.08% alone), administering multiple Δ9-THC doses (10 and 25 mg) that facilitate analysis using the standard Δ9-THC dosing unit (5mg; Volkow and Weiss, 2020), and enrolling an equal number of men and women to explore possible sex differences on study outcomes.

Another strength of the proposed research is that it includes a comprehensive array of impairment measures. Participants will report on their subjective degree of impairment, complete an extensive battery of field sobriety tests and a novel smartphone/tablet-based impairment test (the DRUID application), and perform comprehensive simulated driving scenarios. Greater understanding of how to measure and/or detect impairment from cannabis (when administered alone and with alcohol) is paramount to public health because detecting impairment from cannabis has proven to be far more difficult than for alcohol. The pharmacokinetics of alcohol are linear and predictable, meaning blood and breath alcohol concentrations are reliable indicators of an individual’s current degree of alcohol impairment (Jones, 2010). Moreover, standardized field sobriety tests have been validated to detect alcohol impairment at specific BAC *per se* limits (thresholds that legally constituent impairment); 0.08% BAC is the most common *per se* limit in the U.S. and 0.05% is the most common in Europe. That said, it is unclear whether these established *per se* limits for alcohol impairment should be lowered for individuals who are under the influence of both cannabis and alcohol, as few controlled studies have examined this question.

For cannabis, no behavioral tests have explicitly been developed to detect impairment and standardized field sobriety tests developed for alcohol intoxication have shown limited sensitivity to cannabis impairment in controlled studies (Papafotiou et al., 2005a; Papafotiou et al., 2005b; Spindle et al., 2021). One retrospective study of real-world drivers found that some aspects of certain field sobriety assessments could identify cases of cannabis intoxication compared with controls (Hartman et al., 2016a). However, the sensitivity and validity of these tests have yet to be determined in prospective research; Hartman et al. (2016a) was also subject to potential bias given that other cues of cannabis use (e.g., cannabis odor) were more present in cannabis-intoxicated cases versus controls. In addition, unlike alcohol, concentrations of Δ9-THC in blood and other biologic matrices (e.g., oral fluid) do not correlate highly with cannabis impairment due to the lipophilic nature of cannabinoids and less orderly pharmacokinetics (Huestis, 2007). Despite their limitations, field sobriety tests and blood Δ9-THC *per se* limits are currently the most common approaches for determining cannabis impairment at the roadside. Thus, there is an urgent need to develop and validate novel cannabis impairment detection tools. One potential impairment test is the DRUID which is a smartphone/tablet-based application that has shown good sensitivity to both alcohol (Richman and May 2019) and oral and vaporized high Δ9-THC cannabis impairment (Spindle et al., 2021) in prior studies. The planned study will be a critical next step in evaluating the DRUID, as it will determine whether performance on the DRUID impairment test is predictive of driving impairment caused by individual and co-use of alcohol and cannabis.

Driving simulators have been demonstrated to be accurate proxies for real-world driving and are an effective way to evaluate the impairing effects of various substances under safe conditions (Veldstra et al., 2015; Helland et al., 2016). Collectively, prior studies have revealed that alcohol and/or high Δ9-THC cannabis can impact various facets of driving. Alcohol has reliably been shown to adversely impact lane weaving (or standard deviation of lateral position, SDLP), performance on driving tasks of divided and sustained attention, reaction time, and various other driving performance metrics (e.g., centerline and road-edge crossings). Moreover, alcohol tends to lead to more reckless driving, as evidenced by increasing speed (or more variable speed: standard deviation of speed, SDSP), shortening following distance to lead vehicles, or running red lights or stop signs, all of which may increase the likelihood of accidents. Impairment on these driving performance outcomes is typically observed at a BAC of 0.05% (or lower) and generally increases in a dose-orderly fashion. High Δ9-THC cannabis also negatively impacts simulated driving performance (Martin et al., 2013; Jongen et al., 2016; Irwin et al., 2017; Van Dijken et al., 2020). As with alcohol, high Δ9-THC cannabis reliably increases lane weaving (i.e., SDLP); SDLP has consistently been the most sensitive driving outcome measure following acute high Δ9-THC cannabis exposure (as with alcohol, high Δ9-THC cannabis reliably increases SDLP; Arkell et al., 2020; Marcotte et al., 2022). Likwise, SDLP and other measures of lateral control are particularly impacted by high Δ9-THC cannabis during controlled driving tasks that are more cognitively demanding than regular driving (e.g., divided attention or car following tasks; McCartney et al., 2021; Marcotte et al., 2022; Simmons et al., 2022; Thawer et al., 2022). Unlike alcohol, high Δ9-THC cannabis tends to lead to reduced driving speed (McCartney et al., 2021; Simmons et al., 2022; Thawer et al., 2022). Other driving outcomes are also negatively impacted by high Δ9-THC cannabis (e.g., reaction time, ancillary outcomes on divided attention or car following tasks), albeit in a less consistent fashion than SDLP and speed (McCartney et al., 2021). These inconsistent results are likely due to differences in Δ9-THC doses administered and dosing procedures (e.g., *ad libitum* vs. fixed dosing), and the types of simulations/scenarios used across studies. Though relatively few studies have administered cannabis and alcohol concurrently, co-use of alcohol and high Δ9-THC cannabis has been found to produce additive or synergetic driving impairment, again as evidenced most clearly by measures of lateral control (e.g., SDLP; Hartman et al., 2015; Simmons et al., 2022).

The primary aim of this two-part study is to characterize subjective, cognitive and psychomotor, and driving impairment from oral and vaporized high Δ9-THC cannabis, when administered alone and in combination with alcohol. A secondary aim is to explore possible pharmacokinetic interactions between alcohol and oral/vaporized cannabis. A key aspect of this protocol was the development of custom simulated driving scenarios designed to test important aspects of driving performance that are known to be impacted by alcohol and cannabis exposure. By incorporating different tasks and scenarios that have been independently found to be sensitive to alcohol and cannabis effects, this custom simulation can provide a holistic view of how these substances impact driving performance when taken alone and together. To promote greater standardization and future cross-study comparisons among simulated driving studies, we describe the characteristics of the custom drive scenarios and provide a copy of the simulated drives along with an R script to extract and organize the data (available for download at https://github.com/mdnovak/STISIM_impaired_driving). Below we also describe the study procedures and specific outcome measures, detail our statistical analysis plan, and conclude by commenting on the implications that this research can have for public safety as well as policy and regulatory decisions for alcohol and cannabis.

## 2 Methods

### 2.1 Study design overview

The study is a two-part, randomized, double-blind, placebo-controlled, crossover study examining the acute effects of oral high Δ9-THC cannabis (Part 1) or vaporized high Δ9-THC cannabis (Part 2) and alcohol, alone and in combination, on various pharmacodynamic (e.g., cognitive/psychomotor/driving performance, subjective drug effects) and pharmacokinetic outcomes. All study participants will complete seven separate outpatient sessions where they will consume a cannabis-infused brownie (containing 0, 10, or 25 mg Δ9-THC) or inhale vaporized cannabis (containing 0, 10, or 25 mg Δ9-THC) and a drink that contains placebo or alcohol (alcohol-containing drinks will be calculated to produce a BAC of 0.05%). Placebo drinks contain 1 ml of alcohol, which is placed on top of the beverage immediately prior to dosing and contain an alcohol-soaked hair tie as part of blinding procedures (to mask smell and taste sensory cues). There will also be a positive control session where participants will administer placebo cannabis with an alcohol drink calculated to produce a BAC of 0.08% (selected due to its common use as a *per se* threshold for law enforcement to enforce driving under the influence (DUI) laws). Each session will be conducted at the Johns Hopkins Behavioral Pharmacology Research Unit (BPRU), will last approximately 10 h, and will be separated by at least 1 week to allow for sufficient drug washout. Experimental procedures are approved by the Institutional Review Board of Johns Hopkins University School of Medicine (IRB00290015) and will be conducted in accordance with the Declaration of Helsinki. Part 1 is registered with ClinicalTrials.gov (NCT04931095) and the project was funded by the National Institute on Drug Abuse (R01-DA052295 to TRS).

### 2.2 Inclusion and Exclusion Criteria

Participants will be eligible if they: 1) are 21–55 years old, 2) are in good general health based on screening procedures (see below), 3) are not pregnant or breast feeding (for women), 4) have a body mass index (BMI) between 19 and 38 kg/m^2^, 5) have not donated blood in past 30 days, 6) report using alcohol and cannabis in combination (i.e., “co-use”) at least once in the past year, 7) report ≥ 5 uses of cannabis in the past year, 8) report at least 2 days of binge drinking in the past 90 days (greater than 4 or 5 drinks within 2 h for women and men, respectively), 9) provide a negative urine test for illicit drug use (excluding Δ-9-THC) and a negative breath alcohol test (0% BAC) at screening and before study sessions, 10) report no uses of OTC drugs, supplements/vitamins, or prescription medications that may interfere with participant safety in the past 14 days or within 5 half-lives for that specific drug before study sessions.

Participants will be considered ineligible if they: 1) report psychoactive drug use (aside from cannabis, nicotine, alcohol, or caffeine) in past month at screening, 2) have a history of or current evidence of a medical condition judged by the investigators to impact the safety or validity of the research, 3) have a current Axis I psychiatric condition (MINI for DSM-V), 4) meet criteria for severe alcohol use disorder (MINI for DSM-V), 5) have a CIWA-Ar score >9, 6) have been in treatment previously for alcohol or cannabis use disorder, 7) report using cannabis, on average, more than 2 times/week over past 3 months, 8) have impaired liver function (more than 2x normal range), 9) were enrolled in another clinical trial or received any drug as part of research within past 30 days at screening, and 10) have a Shipley vocabulary score <18 (corresponds to 5th grade reading level).

### 2.3 Study procedures

#### 2.3.1 Recruitment and screening procedures

Research participants will be recruited *via* media advertisements (e.g., newspaper, internet) and word-of-mouth communication. Participants will provide written informed consent and prescription/non-prescription medication use will be obtained. Recent alcohol and drug use will be assessed *via* the 90-day Timeline Follow Back (TLFB) (Sobell and Sobell, 1992). Urine will be obtained and tested for evidence of recent use of the following commonly abused drugs: Amphetamine, Secobarbital, Buprenorphine, Oxazepam, Cocaine, 2-ethylidene-1,5-dimethyl-3,3-diphenyl-pyrrolidine, methylenedioxymethamphetamine (MDMA), methamphetamine, morphine, methadone, oxycodone, phencyclidine (PCP), propoxyphene, nortriptyline, and cannabinoids. Prospective participants will also undergo a physical exam by a physician or nurse practitioner and routine blood tests will be conducted including clinical chemistry, hematology, serology, and serum pregnancy (for females). Other screening assessments include: the MINI for DSM-5 (Sheehan et al., 2010), the Alcohol Use Disorders Identification Test (AUDIT), the Clinical Institute Withdrawal Assessment for Alcohol, revised (CIWA-Ar) (Sullivan et al., 1989; Bush et al., 1998), the brief sensation seeking questionnaire (Stephenson et al., 2003), the Eysenck Impulsivity Questionnaire (Eysenck et al., 1985), and the driving history questionnaire (Kidd and Huddleston, 1994; Owsley et al., 1999). Individuals who are eligible will be scheduled to undergo training on the various performance measures (e.g., DRUID, driving, field sobriety tests, etc.) in order to minimize the potential for practice effects during sessions. Training will occur within 1 week of the first drug administration session. If a participant reschedules their first session, the training session date will also be adjusted so that it is within 1 week of the starting date.

#### 2.3.2 Experimental session procedures

Participants will be instructed to fast the morning of each session and to consume no more than 100 mg of caffeine. Upon arrival, participants will be fed a standard, calorie-controlled, low-fat breakfast and self-report their drug and alcohol use since the last visit. A urine drug test and BAC test will also be conducted at this time. Recent concomitant medication use, including vitamins and herbal supplements, will be recorded before each session and any medication changes will be reviewed by medical staff to ensure the participant is still eligible. An intravenous catheter will be inserted into participants’ forearm vein (non-dominant arm preferred) to enable repeated blood sampling. Participants will complete an 8-min acclimation drive to reorient themselves to the driving tasks and handling of the vehicle (e.g., steering/braking sensitivity) before each session (see Driving Simulator Tasks and Outcome Measures below). Next, participants will complete a “baseline” timepoint of all study assessments (e.g., driving, cognitive performance, etc., see below).

**FIGURE 1:**
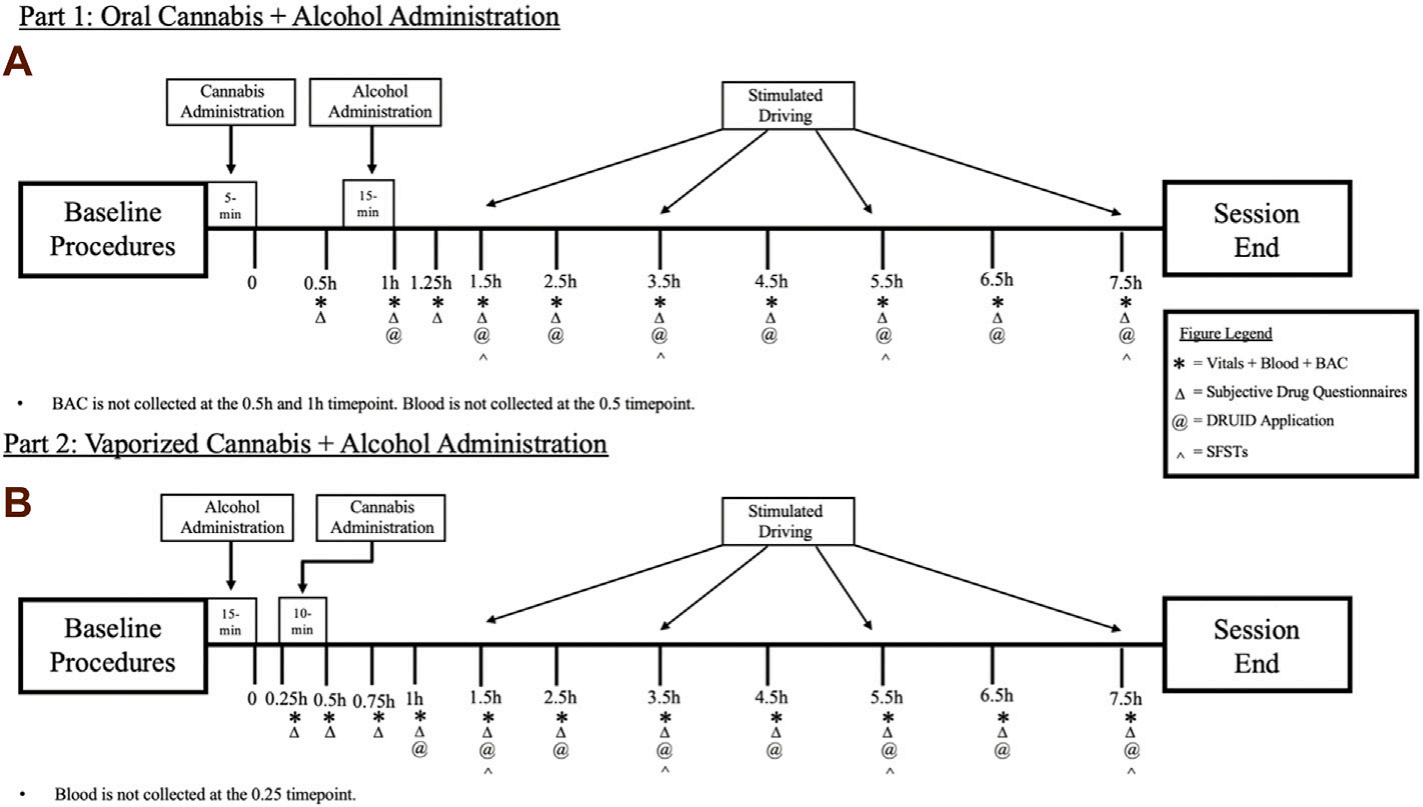
A breakdown of each experimental session for the **(A)** Oral Cannabis + Alcohol (Part 1) and **(B)** Vaporized Cannabis + Alcohol (Part 2) studies. * indicates a collection of vitals (heart rate and blood pressure), blood, and breath alcohol concentrations (BAC). Δ indicates the collection of all subjective questionnaires (drug effect questionnaire; DEQ; subjective high assessment scale; SHAS; biphasic alcohol effects scale; BAES). @ indicates the administration of the DRUID application. ^ indicates the administration of all six Standardized Field Sobriety Tests (SFSTs). All outcomes are collected during baseline procedures.

In the oral dosing study (Part 1), following baseline procedures, participants will first consume a cannabis-infused brownie (containing 0, 10, or 25 mg Δ9-THC; See Table 1). Forty-five min after cannabis ingestion, participants will begin consuming their alcohol or placebo drink (they will consume the drink over 15 min, meaning they finish their alcohol dosing approximately 1 h after brownie consumption). The time at which the brownie is completely consumed (i.e., last bite swallowed) is considered Time “0” by which remaining study assessment time points will be scheduled (see Figure 1A). In Part 2, following identical baseline procedures to Part 1, participants consume their alcohol or placebo drink over 15 min. Twenty min after finishing their drink, participants will self-administer vaporized cannabis containing 0, 10, or 25 mg Δ9-THC within a 10 min period (see dosing procedures below). The end of the 15-min drinking period is considered Time “0” by which remaining study assessment time points are scheduled (see Figure 1B). After drug administration, participants will complete study assessments again at 15–60 min intervals until they are discharged (see Figures 1A,B).

**TABLE 1:** Study drug and dose conditions.

Part 1		Part 2	
Oral cannabis (mg Δ9-THC)	Alcohol (BAC %)	Vaporized cannabis (mg Δ9-THC)	Alcohol (BAC %)
0	0	0	0
10	0	10	0
25	0	25	0
0	0.05	0	0.05
10	0.05	10	0.05
25	0.05	25	0.05
0	0.08	0	0.08

BAC, 0.08 serves as the study’s positive control in Part 1 and Part 2.

### 2.4 Drug preparation and dosing procedures

High Δ9-THC and placebo cannabis were obtained for this study from the National Institute on Drug Abuse (NIDA) Drug Supply Program. The cannabis contains: 18.16% Δ9-THC, <0.03% cannabidiol (CBD), 0.11% tetrahydrocannabivarin (THCV), 0.38% cannabinol (CBN), 0.24% cannabichromene (CBC), and 0.38% cannabigerol (CBG). This cannabinoid profile is comparable to high Δ9-THC cannabis sold in dispensaries and high Δ9-THC plant material seized by law enforcement in the United States (Vergara et al., 2017; ElSohly et al., 2021). The placebo cannabis contains: <0.01% Δ9-THC, CBD, CBN, THCV, CBC, and CBN. Participants will be given the same quantity of cannabis in each experimental condition. Using this batch of cannabis, 137.8 mg will be used to achieve a 25 mg Δ9-THC dose, 55.1 mg of active cannabis and 82.7 mg of placebo cannabis will be used to achieve a 10 mg Δ9-THC dose, and 137.8 mg of placebo cannabis will be used for placebo conditions.

In Part 1, cannabis brownie preparation will consist of: (1) grounding cannabis into a fine powder; (2) baking cannabis at 250°C so that tetrahydrocannabinolic acid (THC-A) will decarboxylate to Δ9-THC, and (3) mixing decarboxylated cannabis with a commercial brownie batter, along with other ingredients (eggs, vegetable oil, etc.). Each brownie will be baked individually to ensure accurate dosing (precisely weighed amounts of ground cannabis will be included in each brownie). In prior studies, we have ensured that these methods reliably yield targeted Δ-9-THC doses (Vandrey et al., 2017; Spindle et al., 2021). Active and placebo brownies will be made with the same quantity (g) of cannabis to assist with blinding.

In Part 2, participants will self-administer vaporized cannabis using the Mighty Medic vaporizer (Storz-Bickel, Tuttlingen, Germany), a handheld commercial vaporizer designed specifically for the delivery of cannabis and Δ9-THC. The Mighty Medic is an approved medical device in the European Union, Canada, and Israel. Precisely weighed amounts of cannabis are placed in disposable dosing capsules (or “pods”), heated at 204°C, and participants inhale the resulting vapor. In each Part 2 session, participants will inhale the entire contents of a dosing capsule in an *ad libitum* manner; use of a paced puffing procedure in a previous study resulted in more coughing and symptoms of hyperventilation compared with *ad-libitum* puffing. In prior studies (Spindle et al., 2018; Spindle et al., 2019; Arkell et al., 2020), we have shown that our *ad-libitum* dosing procedure results in consistent dose delivery across individuals and sessions and produces dose-orderly drug effects. New mouthpieces and pods will be used in each session to prevent contamination from prior doses. The BPRU pharmacy will store, prepare, and dispense cannabis for both studies.

Alcohol drinks with doses targeted to produce BACs of 0.05% or 0.08% will be self-administered by participants. Three alcohol drinks (each containing a third of the total active alcohol dose) will be made by mixing an exact amount of 95% grain alcohol (minus 1 ml for blinding, see below) with a non-caloric sweetened beverage (i.e., sugar-free Cherry Kool Aid) to achieve a volume of 4 oz each (12 oz total). Placebo drinks will be prepared by floating 1 ml of alcohol on top of the 4 oz flavored beverage immediately prior to dosing as part of blinding procedures; to further facilitate blinding, an alcohol-soaked hair tie will be placed around each glass, delivering a strong alcohol odor for placebo and active drinks. Drinks will be consumed over a 15-min period. Specifically, participants will ingest each drink within a 5-min time frame and will wait until the next 5-min window before administering the next drink. Alcohol doses for each participant will be calculated with the Computerized Blood Alcohol Calculator (CBAC©) (Addiction Research Foundation; Fisher et al., 1987). The amount of alcohol needed to produce a given BAC is calculated by factoring in sex, age, height, weight, calculated total body water, and time spent drinking. In prior alcohol dosing studies, we have confirmed that the CBAC reliably produces specific BACs (e.g., 0.05% or 0.08%) across the lifespan in men and women with a range of alcohol use and drinking patterns (Uhart et al., 2013; Weerts et al., 2017; McCaul et al., 2018).

### 2.5 Outcome measures

All study outcomes described below will be assessed at baseline and at specified timepoints after drug exposure (Figure 1). The proposed timepoints were designed to capture the full time course of drug effects for each route of cannabis administration.

### 2.6 Simulated-driving performance: Overview

**FIGURE 2:**
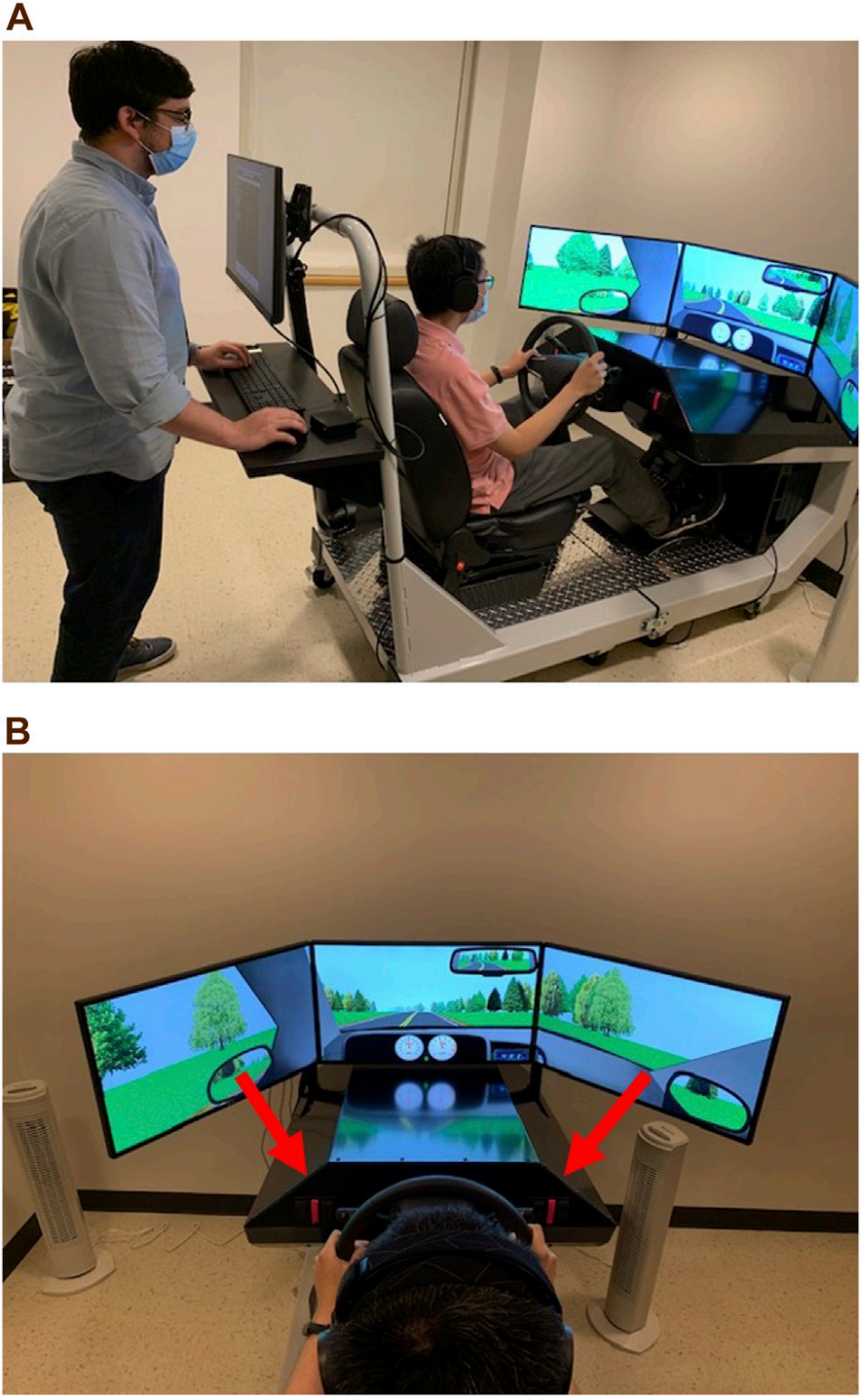
STISIM M40000-R Drive driving simulator with a sample driving scenario. **(A)** depicts the operator interface where session administrators (left) set preselected driving scenarios. **(B)** illustrates the three-screen display and arrows indicate the divided attention task’s buttons (pink tape).

The STISIM M40000-R Drive driving simulator (software: STISIM Drive version 3.14; Figure 2) will be used in this research to assess driving performance (System Technology, Inc. Hawthorne, CA). The simulator consists of three video monitors that provide a 135° field-of-view, as well as a car seat, steering wheel, turn signal lever, accelerator, and gas and brake pedals. The participant can fully control the speed and maneuvering of the vehicle. Seat and monitor positioning, monitor size, turning signals, responsiveness of gas and brake pedals and steering wheel, and viewing angles were all engineered to conform to automotive ergonomics and accurately simulate the experience of driving an actual vehicle. Prior research has independently confirmed that STISIM Drive® simulators are valid reflections of real-world driving conditions (Shechtman et al., 2009; Mayhew et al., 2011).

The fully interactive driving simulations created for this research feature a range of routine and non-routine events, including driving through residential, rural, construction zone, and metro sections, as well as two controlled driving scenarios. During routine driving sections, participants will encounter various events and challenges a driver may encounter on the road such as signal lights (some requiring turning maneuvers) and stop signs, other vehicles that must be navigated around, pedestrians, or other similar challenges. The two controlled scenarios include a Car Following Task and a Divided Attention Task. Data are collected during the routine driving sections at specific events that may be impacted by alcohol and cannabis intoxication (e.g., response to unexpected stimuli to avoid collisions, interactions with stoplights) and data is collected throughout the two controlled scenarios. Car following and divided attention tasks have shown good sensitivity to alcohol (Freydier et al., 2014; Miller et al., 2020; Garrisson et al., 2021) and cannabis impairment (Arkell et al., 2020; Marcotte et al., 2022) in prior studies because these two controlled scenarios test key aspects of driving performance known to be affected by these drugs (e.g., divided/sustained attention, processing speed, psychomotor ability, maintaining lane position, speed, and distance to other cars; Thawer et al., 2022). Beyond these specific events and scenarios, additional summary variables of interest are passively collected throughout the drives (see below).

A total of ten unique drives were created for this project to avoid potential practice/learning effects (e.g., memorizing avoidance scenarios). Drives differ by the order with which events and scenarios are presented and by the type of accident avoidances that occur, but the drives are otherwise identical so that each version will produce comparable results. For example, the length of each drive, the environments encountered, and key drive features are the same for each simulation. Other vehicles in the simulations are randomly generated, which further increases the novelty of each drive. Beyond mitigating practice/learning effects, the different drive iterations also ensures that each drive is novel to the participant on a given session day; at each driving timepoint, participants are randomized to receive any of these 10 drives, but the same drive will not occur twice in a given session. Each drive is approximately 18 miles long and takes about 25 min to complete. All 10 versions of the driving simulation (as well as an R script to extract and organize the data) are available to download at: https://github.com/mdnovak/STISIM_impaired_driving. Drives will be performed at baseline and again 1.5, 3.5, 5.5, and 7.5 h after Time 0 (see Figure 1).

### 2.7 Driving simulator tasks and outcome measures

#### 2.7.1 General driving summary data

Each simulated drive collects a series of variables associated with participants’ global driving behaviors throughout the entire drive. These variables can be classed into 4 categories: Accidents (number of collisions, pedestrians hit, and off-road accidents), Rule-Following (number of missed stop signs, stops at red lights, and illegal turns), Speed (number of speed exceedances, total run length and speed, and percentage of time driven over the speed limit), and Lateral Movement (number of centerline crossings, road edge excursions, and percentage of time driven out of lane; Dahlgren et al., 2020). These variables are collected continuously and independent of the of programmed driving events.

#### 2.7.2 Baseline acclimation drive

Prior to the start of each session’s baseline procedures, participants will complete an acclimation drive to reorient themselves to the driving tasks and handling of the vehicle (e.g., steering/braking sensitivity) that will include: a 3-min stopping and turning segment, a 1-min car-following task, a 1-min divided attention task, and a 3-min normal driving segment that includes one crash avoidance scenario. No data will be collected from this drive.

#### 2.7.3 Car-following task

**FIGURE 3:**
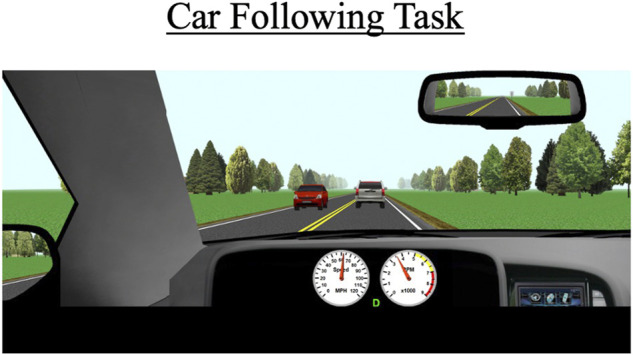
The car-following task occurs behind a white SUV over a duration of 5 min. The primary outcomes for this scenario are standard deviation of lateral position (SDLP), the car’s modulus, and the coherence to the task.

During each drive, participants will perform a 5-min car-following task. This task commences when a vehicle appears about 300 ft in front of the participant’s vehicle (Figure 3). Participants are instructed to follow the lead vehicle and to try to maintain a constant distance (headway) to it. The lead vehicle accelerates and decelerates every 30 s in a sinusoidal manner. The lead vehicle’s speed fluctuates between 50 and 70 mph (80 and 110 km/h). The task occurs on a two-lane road; cars periodically drive by in the opposite lane but there are no cars aside from the lead vehicle in the participant’s lane.

The primary outcome measures for this task include SDLP (a composite measure of lane weaving, swerving, and over-correcting), car-following modulus, response delay, and coherence. The modulus variable indicates how well the participant’s speed matches the lead vehicle’s speed; a value of 1 represents a perfect match, values greater than 1 indicate the participant generally drove faster than the lead vehicle, which indicates tailgating behavior, and values under 1 indicate the participant tended to drive slower than the lead vehicle. Car-following delay represents the amount of time it takes for the participant to respond to speed changes by the lead vehicle. Car-following coherence demonstrates how well the participant’s overall data matches that of the lead vehicle; coherence is expressed as a correlation from 0–1 (0 = no correlation between participant’s and lead vehicle’s data; 1 = perfect correlation between the two vehicles’ data).

#### 2.7.4 Divided attention task

**FIGURE 4:**
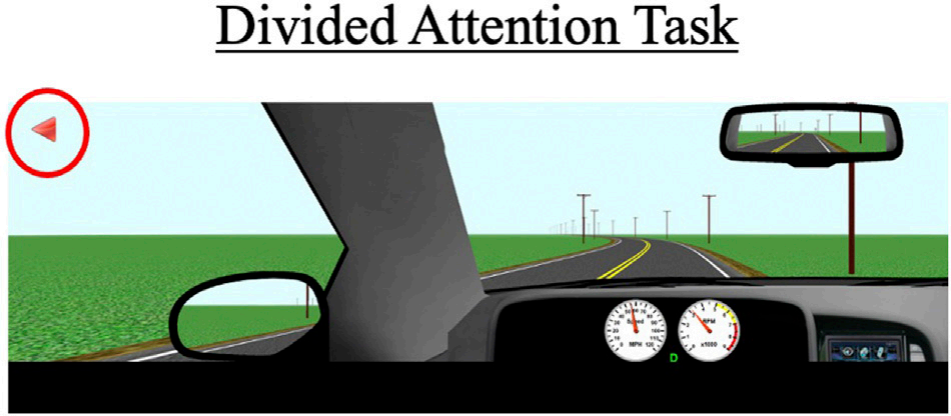
The divided attention task with arrows (red circle) that appear on the left and right monitor displays. Participants must press the corresponding buttons (see Figure 2) within a 5-s timeframe to receive a “correct” response. The primary outcomes for this scenario are SDLP, standard deviation of speed (SDSP), the percentage of correct responses, and the average reaction time to the 20 trials.

During this task, participants drive for approximately 3 miles on a two-lane road with no other cars present. The posted speed limit is 55 mph (88 km/h). Periodically, symbols appear in one of four designated quadrants on the left or right monitor. Participants are instructed to respond when they see these symbols by pressing buttons located adjacent to the steering wheel (see Figure 4); a symbol appearing on the left monitor would prompt a left button response and a symbol appearing on the right monitor would prompt a right button response. A total of 20 trials will be presented to the participant, with each symbol appearing for a maximum of 5 s and disappearing once a correct or incorrect response is recorded. Participants are also instructed to try to maintain a constant lane position throughout the task. Primary outcome measures include correct responses to the presented symbols (out of 20), mean reaction time to respond to the symbols, SDLP, and SDSP (a measure of speed variability).

#### 2.7.5 Control straightaway segment

Following completion of the car-following task, participants continue driving on the same straight roadway for another 2 miles. The posted speed limit is 70 mph (110 km/h). This stretch of roadway is identical to the roadway used for the car-following task. The purpose of the stretch is to assess driving performance in the absence of any additional cognitive demands, thus serving as an important comparator to performance data collected under increased cognitive load (i.e., during the car following and divided attention tasks). Primary outcome measures during this segment are SDLP and SDSP.

#### 2.7.6 Crash avoidance events

**FIGURE 5:**
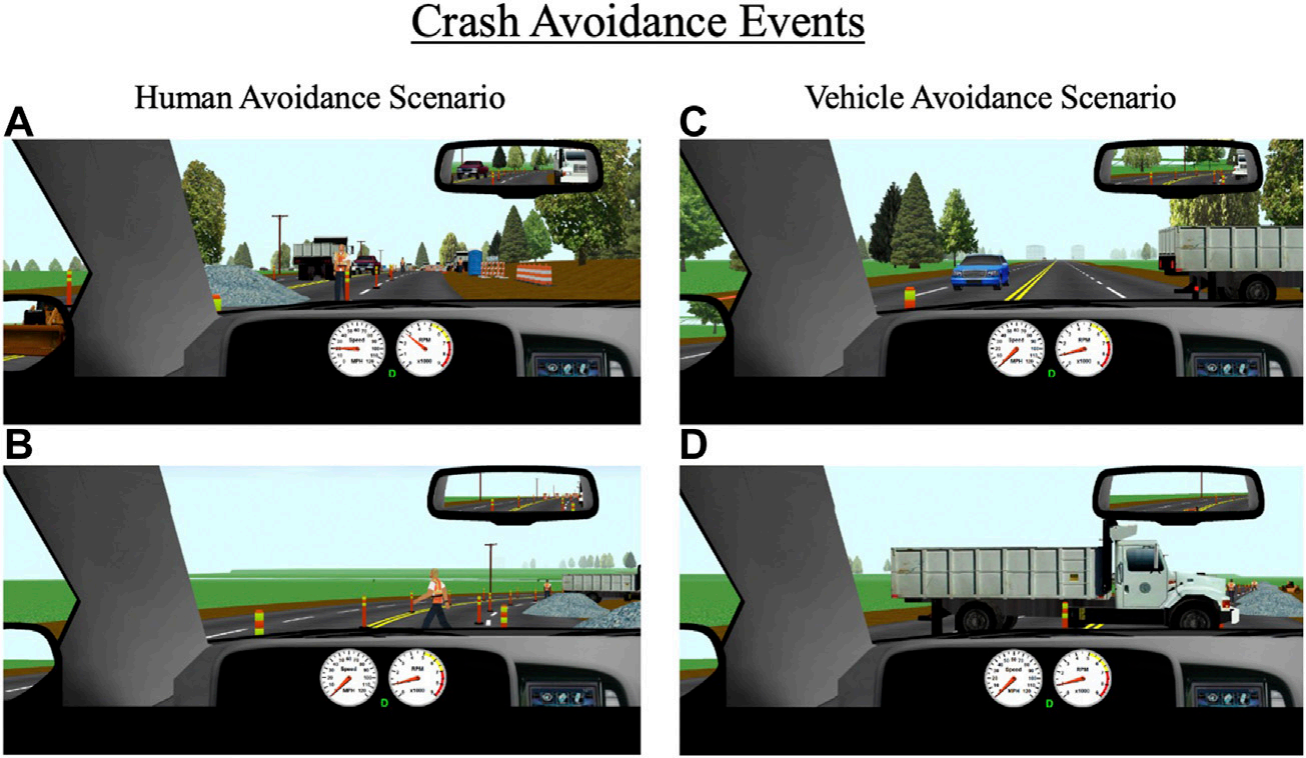
Crash avoidance events that occur throughout the drive for **(A,B)** human avoidance scenarios, and **(C,D)** vehicle avoidance scenarios. The primary outcomes for these events are collisions, reaction time to the event, and time-to-collision with the event.

As mentioned above, participants encounter a variety of environments throughout the 25-min drive such as residential neighborhoods, urban and metropolitan areas, rural areas, and construction zones. Each of the 10 drives contain three unique pre-determined crash avoidance scenarios interspersed throughout these areas. Each crash avoidance occurs in a roadway segment with a posted speed limit of 35 mph (55 km/h) and is designed to be unexpected (e.g., pedestrian or deer suddenly walking into the road, vehicle backing out into the road). The three unique avoidances in each drive are set to a different difficulty level (i.e., “easy”, “medium”, and “hard”). This allows for crash risk from using alcohol, cannabis, and alcohol and cannabis in combination to be assessed for avoidance scenarios of a range of difficulties (Figure 5).

Avoidance events are programmed to trigger based on the driver’s headway time from the object (e.g., pedestrian); easy, medium, and hard avoidances are set to headway times of 3.0, 2.5, and 2.0 s, respectively. Setting all avoidances based on headway time ensures a standard duration to react to a given avoidance (regardless of their approaching speed) and allows for direct comparisons across different types of avoidances. All avoidances were designed in ways that force participants to make a response and reduce the likelihood that they can ignore the avoidance. For example, for several avoidances, participants are constrained to a single lane at the time of appearance. The primary outcome measures for the crash avoidances are reaction time and time-to-collision (TTC) with the object. Reaction time to each avoidance is based on the participant’s initial response to the object after it appears, which could be a braking or steering response. TTC is calculated based on the participant’s distance from the object at the time of their initial response and their current speed at the time of their response (i.e., distance/speed = TTC).

#### 2.7.7 Stop light interaction tasks

**FIGURE 6:**
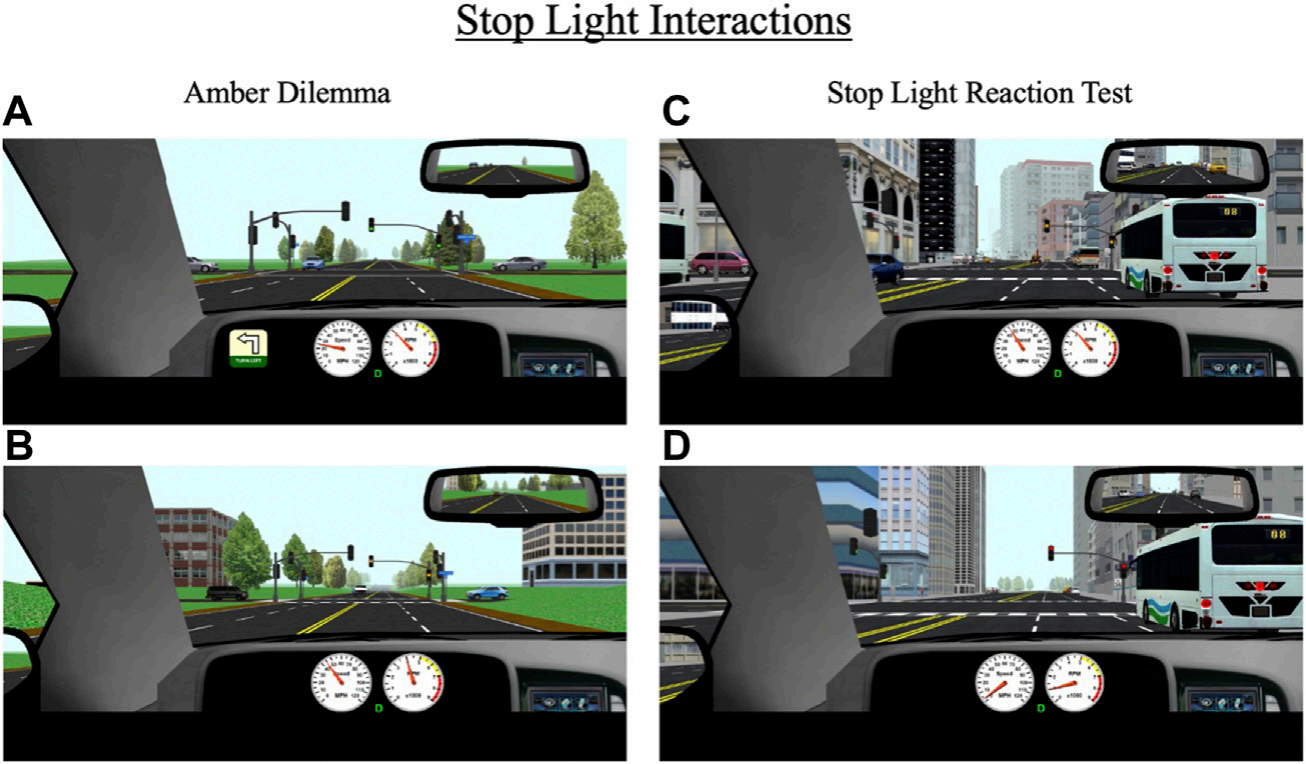
Stop light interactions for **(A,B)** amber dilemmas and **(C,D)** stop light reaction test that occur throughout the drive in rural and urban environments. During amber dilemmas, participants will approach a green light **(A)** that will change to yellow **(B)** 3.5 s from the intersection. During the stop light reaction test, participants will approach a green light that will change to yellow **(C)** 5.5 s from the intersection and then to red **(D)** 3.5 s after the yellow transition. The primary outcomes for these events are response to the traffic signal (i.e., stop or go through the light), reaction time to the signal light, and if participants stop, their response time to a green signal light.

Over the course of each drive, participants encounter eight stoplights, some of which are programmed to create two scenarios of interest: an amber light dilemma or a stoplight reaction test. The specific lights where these scenarios occur differ across the 10 drives. An amber light dilemma is a scenario in which a traffic light turns yellow as a driver approaches it and they must decide whether to stop or continue driving. This scenario has been conceptualized as a model of risk-taking; individuals who choose to continue driving in response to a yellow light risk running a red light, which may result in a traffic violation or crash in the intersection while those who choose to stop at the light may be considered more risk-averse (Palat and Delhomme, 2016; Pathivada and Perumal, 2019). During each drive, participants encounter two “true” amber dilemmas (one in a rural area and one in an urban area), meaning there is not necessarily a correct response; they can safely proceed through the light without receiving a penalty (e.g., a ticket) or can safely come to stop. All yellow lights are programmed to last 3.5 s, which is consistent with Maryland state traffic laws. For amber dilemmas, the stoplights are programmed to turn yellow when a participant’s vehicle is 3.5 s (in terms of headway time) from the light, thus maximizing the indecision zone (Figures 6A,B).

The second stoplight scenario, the stoplight reaction test, is similar to the amber dilemma in that a traffic light turns yellow as a driver approaches. However, in this scenario, the light turns yellow much earlier (at a headway time of 5.5 s) and the correct response is for the participant to stop (Figures 6C,D). If they proceed through the light, they receive a ticket. Each drive contains one stoplight reaction test. The primary outcome measures for amber light dilemmas and stoplight reaction tests are reaction time to respond to the yellow light change (either gas or brake response) and whether the participant proceeded through or stopped at the light. For the stoplight reaction test, an additional outcome measure is reaction time to begin driving again in response to a green light (assuming the participant did not run the yellow light).

#### 2.7.8 Composite drive score

As in Marcotte et al. (2022), primary variables from various individual components of the drives will be integrated to produce a single value indicative of overall driving performance (or a “Composite Drive Score”). Composite Drive Scores will be calculated for each post-dosing drive and compared to the baseline drive (i.e., prior to cannabis and alcohol dosing) within a given session. In order to calculate the Composite Drive Score, z-scores will be established based on baseline driving performance, using the mean and standard deviation of each score (i.e., the primary and secondary outcomes) for each participant across all seven baseline drives (the baseline Composite Drive Score for each participant will have a mean z-score of 0, with a standard deviation of 1). Higher scores on this measure will indicate worse driving performance. For this study, the Composite Drive Score will be comprised of primary and secondary driving outcomes from the Car-Following Task (e.g., SDLP, Coherence), Divided Attention Task (e.g., SDLP, SDSP, percent correct responding), and crash avoidances (e.g., Reaction Time, Time to Collision).

### 2.8 Computerized cognitive and psychomotor measures

The DRUID application (Impairment Science Inc., Cambridge, MA, United States), which has been shown to be sensitive to both alcohol (Richman and May 2019) and cannabis impairment (Spindle et al., 2021), will be used to measure cognitive and psychomotor performance. The DRUID application includes four 30–45 s tasks that measure reaction time, decision-making, hand-eye coordination, and time estimation under conditions of divided attention in addition to a balancing task. On Task 1, shapes (either a square or circle) flash on the screen; one shape is designated as the target shape and the other the control shape. Participants are asked to touch the screen where the target shape appears and to touch the top of the screen when the control shape appears. Reaction time in touching the screen is assessed along with the number of errors. On Task 2, participants estimate when they feel 30 s have passed by pressing a button on the screen and, while waiting to press the button, they touch the screen where shapes briefly flash. Reaction time to the stimuli and accuracy of time estimation are measured. On Task 3, participants try to keep their finger on a circle that moves randomly around the screen while also counting the number of squares that flash on the screen. For Task 4, participants stand on one leg for 15 s while holding the iPad in their same hand, then following completion, perform this task on the opposite leg (with the iPad in the other hand); DRUID accesses data from an accelerometer located in the iPad to measure stability and balance during this task. Performance data from each of the four tasks is integrated using a statistical algorithm to yield a Global Impairment Score, the primary outcome measure for the DRUID. The DRUID battery will be given at baseline and again at various timepoints up to 7.5 h post-dosing (see Figure 1). The DRUID will be administered immediately after driving.

### 2.9 Standardized field sobriety tests

A battery of field sobriety tests commonly used by law enforcement to assess impairment from drugs/alcohol at the roadside will be administered to participants by research staff. Research staff were formally trained on how to administer these tests alongside active-duty police officers during a three-day in-person instructional course held by the Baltimore County Police department, normally only offered to police personnel. In addition, a retired certified Drug Recognition Expert (DRE) for the Maryland State Police provided supplementary training and will periodically attend study sessions to confirm study staff maintain fidelity to the task procedures. A total of six field sobriety tests are administered: the Horizontal Gaze Nystagmus (HGN), which will include the Vertical Gaze Nystagmus (VGN), the Walk-and-Turn (WAT), the One-Leg Stand (OLS), Lack of Convergence (LOC), the Modified Romberg Balance (MRB), and the Finger-To-Nose (FTN). The HGN, WAT, and OLS are the core battery of tests known as Standardized Field Sobriety Tests (SFSTs) that have been shown to be sensitive to alcohol intoxication (Stuster and Burns, 1998); the remaining tests are also commonly used by law enforcement but are not part of the core SFST battery. While participants perform each test, the task administrator will look for distinctive behavioral indications (i.e., “clues” or “cues”) that may indicate impairment, which will be scored as either present (1) or not present (0); in general, a greater number of clues/cues on a given task suggests a greater likelihood of impairment. Note that the SFST battery includes validated “clues” of impairment while the remaining tests include “cues” of possible impairment that have not been validated *via* formal research.

For the HGN, the task administrator holds a stimulus 12–15 inches from the participant’s face to assess involuntary jerking of the eyes *via* six possible clues: lack of smooth pursuit (left and right eye), distinct and sustained nystagmus at maximum deviation (left and right eye), and onset of nystagmus prior to 45° (left and right eye). Participants are instructed to stand with their feet together, with hands at their sides, hold their head still, and follow the motion of the stimulus with their eyes only. Each eye is checked for each clue, beginning with the participant’s left eye, and all checks are performed twice. Lack of smooth pursuit is performed by passing the stimulus in front of the eyes, from the far tip of each eye to the other (not far enough that the participant would have to move their head), over the course of 2 seconds. The presence of jerking during the smooth passing of the eyes is a clue. Distinct and sustained nystagmus at maximum deviation is performed by passing the stimulus to the far side of the participant’s eye (until little to no white is showing in the corner of the eye at maximum deviation). The presence of jerking at maximum deviation is counted as a clue. Onset of nystagmus prior to 45° is assessed by slowly moving the stimulus from the center starting position to the edge of the participant’s left or right shoulder. The movement should take approximately 4 s and any jerking that occurs prior to 45° is counted as a clue. The presence of four or more of clues on the HGN indicates the individual is impaired (Stuster and Burns, 1998; NHTSA, 2017).

For the VGN, the task administrator holds a stimulus 12–15 inches from the participant’s face, parallel to the floor. The stimulus is raised until the subject’s eyes are as elevated as possible and is then held in place for 4 seconds. The process is repeated twice. The presence of jerking is counted as a clue (Citek et al., 2003).

LOC is assessed by performing two circular motions with the stimulus before approaching the bridge of the participant’s nose. Failure for the eyes to converge (i.e., crossing of the eyes) is considered a cue of possible drug/alcohol impairment. Visual deficits for each participant will be addressed during screening and will be excluded from analyses of the HGN, VGN, and/or LOC task if visual deficits persist. Additionally, for all eye-related measures, prescription eyeglasses will be removed prior to the test.

When administering the HGN, VGN, and LOC, task administrators will use the ToxOptix X3 (ToxOptix, Austin, TX) as the stimulus. This is a specialized pen-shaped tool designed specifically for administering these field sobriety tests. The ToxOptix X3 guides the administrator on proper timing for each individual test *via* a series of vibrations (e.g., the device vibrates to indicate when the administrator should hold the stimulus in place during the HGN). Overall, use of this device can better standardize task administration relative to a conventional stimulus (e.g., a pen).

For the WT, participants are asked to take nine heel-to-toe steps down a straight line (clearly marked on the ground), starting with the right foot. On the ninth step, participants will turn around, pivoting on the left foot using a series of small steps, and take nine heel-to-toe steps back in the opposite direction. There are eight possible clues: failure to balance during the instructional phase, initiating movement prior to instruction, stopping during the task, not touching feet heel-to-toe (a separation distance of 6 inches or greater), stepping off of the line, using their arms for balance, an improper turn, and an incorrect number of steps in either direction. Detection of two or more clues indicates drug/alcohol impairment (Stuster and Burns, 1998; NHTSA, 2018).

For the OLS, participants are asked to raise one foot 6 inches off the ground and count until they are told to stop; they will be stopped by the task administrator after 30 s. There are four possible clues on the OLS: swaying while balancing, using arms for balance, hopping on one leg, and putting their foot down prior to completion. Failure on two or more clues indicates drug/alcohol impairment (Stuster and Burns, 1998; NHTSA 2018).

For the MRB, participants are asked to stand with their feet together and arms at their side, with their eyes closed and head tilted back, and to estimate the passing of 30 s. There are four possible cues of impairment: incorrect time estimation (+/− 5 s), eyelid tremors, body tremors, and swaying.

For the FTN, participants are asked to stand with their feet together and arms at their side, with both index fingers pointed at the ground and their eyes closed and head tilted back. They are then instructed to touch the tip of their index finger to their nose in a specified order. Possible cues of impairment include: failure to follow the instructed order of nose presses and number of failed attempts at touching the tip of their finger to their nose.

The total number of clues/cues of impairment detected will be summed to produce an overall score for each task (our primary outcomes), with higher scores indicating greater impairment; another outcome is the cumulative number of clues/cues of impairment across all tests. Participants are video recorded while performing the field sobriety tests to allow for a second person to score the tests at a later time; agreement between these two coders will be calculated and individual discrepancies between coders will be resolved by a third coder. Eye-related tests are filmed *via* a GoPro mounted on the administrator’s forehead. To further ensure proper technique is followed by task administrators and that clues/cues of impairment were scored correctly, the aforementioned retired DRE who helped train the research staff will score a subset of the recorded tests (approximately 25%, chosen at random). All SFSTs will be given at baseline and at various timepoints after drug administration (up to 7.5 h post-dosing). Each of these tests will be administered immediately after driving.

### 2.10 Subjective drug effects

A modified 25-item Drug Effect Questionnaire (DEQ; Spindle et al., 2018; Spindle et al., 2021) will be administered that includes items assessing: positive (e.g. “like drug” and “pleasant drug effect”) and negative subjective drug effects (e.g. “unpleasant drug effect” and “anxious/nervous”), behavioral/mood states related to acute high Δ9-THC cannabis exposure (e.g. “high”, “paranoid”, and “hungry/have munchies”), and participants’ perceived level of impairment (e.g. “trouble with memory”, “difficulty with routine tasks”, “confidence in driving ability”). Each item will be presented individually on a 100 mm visual analog scale (VAS) with a horizontal line anchored from 0 (“not at all”) on the left to 100 (“extremely”) on the right. The one exception is the question, “Would you drive in your current state,” which requires a yes or no response.

The Subjective High Assessment Scale (SHAS) (Martin et al., 1993) and the Biphasic Alcohol Effects Scale (BAES) (Schuckit, 1980) will be administered to assess alcohol-specific subjective effects. For the SHAS, participants rate alcohol effects on a 100 mm VAS (0 or “not at all” to 100 or “extremely”). Example items include: muddled/confused, slurred speech, dizzy, drunk, distorted sense of time, and difficulty concentrating. Items are summed to produce an overall score (the primary SHAS outcome). The BAES measures stimulant (e.g., energized, excited, stimulated) and sedative (e.g., inactive, sedated, sluggish) effects of alcohol with 14 items. Participants rate each item on a scale from 0 (“not at all”) to 10 (“extremely”). Overall stimulant and sedative scores are calculated from the individual items (two primary BAES outcomes). These questionnaires, along with the DEQ, will be given at baseline and at various timepoints after drug administration (see Figure 1). These questionnaires will be administered immediately before driving.

After each drive, participants will answer two questions to assess their perceived driving performance: “how much did the study drugs affect your driving?” (0 [not at all] to 100 [extremely]) and “how well did you drive?” (0 [not well at all] to 100 [extremely well]).

### 2.11 Physiological measures

Heart rate (HR), systolic blood pressure (SBP), and diastolic blood pressure (DBP) will be measured in the seated position with an automated device at baseline and at regular intervals after drug administration.

### 2.12 Breath alcohol procedure

The Alco-Sensor IV breathalyzer will be used to measure participants’ BAC throughout the sessions. This will be used to ensure that participants reach the target BACs and to monitor BAC levels over time. BAC readings will not be viewable to participants or study staff to maintain dose blinding and will be measured by a nurse who will not administer any of the aforementioned tests.

### 2.13 Blood specimens

Whole blood specimens will be collected using 6 ml gray-top vacutainer tubes and stored frozen at −80°C until sent to a designated laboratory for analysis. Blood collection timepoints for each route of cannabis administration (oral and vaporized) are depicted in Figure 1. Concentrations of Δ9-THC and the two primary metabolites of Δ9-THC (11-OH-Δ9-THC; Δ9-THC-COOH) will be determined using LC-MS/MS.

### 2.14 Data presentation and statistical analysis plan

Power analyses for each primary outcome were conducted in R, using the SIMR package (Green and Macleod, 2016), which estimates power for linear mixed models by fitting a regression model multiple times under different scenarios, or simulations (100 simulations were performed for each power calculation). Effect sizes (Cohen’s d) and data (means and SDs) were obtained from prior alcohol/cannabis co-use studies (Hartman et al., 2016a) and/or prior studies conducted in our laboratory (Spindle et al., 2018; Spindle et al., 2021) that administered the proposed doses of oral/vaporized cannabis or alcohol alone. Overall, all power analyses revealed that 32 participants (16 men/16 women) in Part 1 and Part 2 would provide sufficient power (≥ 0.80 power for all 100 simulations) to test the study hypotheses stated.

For each outcome, descriptive statistics (means and standard deviations) will be generated. Linear mixed models will be used to test for differences across experimental conditions. Hypothesis tests will be two-sided, and significance will be set at *p* < 0.05. Factors entered into these models will include: Time (number of levels will differ depending on outcome variable), Alcohol Dose (0%, 0.05%, and 0.08%), Cannabis Dose (0, 10, and 25 mg Δ9-THC), and Sex (male or female). Time, alcohol dose, and cannabis dose will be within-subject factors (i.e., repeated measures); sex will be a between-subject factor. Maximum concentration (C_max_) and area under the curve (AUC) for Δ9-THC and Δ9-THC metabolites (11-OH-Δ9-THC; Δ9-THC-COOH) will be entered as covariates. Holm-Bonferroni corrections will be used to control for multiple comparisons. Data will be inspected for normality (skewness, kurtosis, outliers) and, where necessary, data transformations will be applied. Missing data is expected to be rare, as tasks and instruments are computerized, and field sobriety tasks will be video recorded. Additionally, if a participant presents elevated concentrations of Δ9-THC or Δ9-THC metabolites at baseline, and these values do not increase following active drug administration, this participant will be excluded from analysis.

For Part 1 and Part 2, a priori planned comparisons will be conducted to compare mean peak change-from-baseline scores for each outcome for: (1) alcohol alone (BAC 0.05%) to alcohol with cannabis (BAC 0.05% + 10 mg Δ9-THC and 25 mg Δ9-THC); and 2) cannabis alone (10 mg Δ9-THC and 25 mg Δ9-THC) to cannabis with alcohol (BAC 0.05% + 10 mg Δ9-THC and 25 mg Δ9-THC). Additionally, planned comparisons will compare impairment (e.g., DRUID scores, composite drive scores) between cannabis with alcohol (BAC 0.05% + 10 m g Δ9-THC and 25 mg Δ9-THC) to the positive control condition (alcohol alone: BAC 0.08%). Correlations will be conducted to assess the relations between DRUID and driving performance. Sensitivity and specificity analyses will be conducted to characterize the effectiveness of the DRUID for determining driving impairment. Specifically, we will plot the sensitivity (true positive rate) and specificity (true negative rate) of the DRUID at identifying impaired driving by comparing various DRUID cutoffs to established cutoffs for driving simulator data that are indicative of clinically meaningful impaired driving in real-world settings (e.g., SDLP change-from-placebo ≥ 2.4 cm; Jongen et al., 2016). Sensitivity and specificity will be calculated as follows: sensitivity (100 × [TP/(TP + FN)]) and specificity (100 × [TN/(TN + FP)]).

## 3 Comments

Controlled human laboratory studies are a valuable method for developing a detailed understanding of the acute impairment profile of alcohol, cannabis, and co-use of both substances. However, studies on alcohol and cannabis co-use are scarce and the few that have been conducted have typically administered smoked cannabis. Oral and vaporized cannabis products are increasing in popularity and these alternative forms of cannabis may produce stronger and/or more prolonged effects compared with smoked cannabis. However, there is presently limited data on the acute effects of co-use of alcohol and oral/vaporized cannabis, and the one study previously published on alcohol and vaporized cannabis co-use showed a significant increase in Δ9-THC and its metabolites in the combined drug condition versus cannabis alone (Hartman et al., 2015). This two-part randomized crossover clinical laboratory study will extend prior research by characterizing the acute impairing effects of alcohol and oral and vaporized high Δ9-THC cannabis, both alone and together, across multiple ecologically relevant doses. Assessments include a state-of-the-art driving simulator, a battery of field sobriety tests and cognitive and psychomotor performance tasks, and subjective drug effect instruments.

This study was designed to maintain a high degree of scientific rigor and experimental control, while also providing ecologically valid and generalizable data. Participants will complete 7 double-blind, double-dummy outpatient sessions where they will administer placebo or active cannabis (10 or 25 mg Δ9-THC) and a placebo drink or alcohol drink calculated to produce a BAC of 0.05%; there is also a positive control session with placebo cannabis and alcohol at a target BAC of 0.08%. The double-dummy design in which participants always receive both an experimental beverage (containing alcohol or placebo) and cannabis (active or placebo) will help control for expectancy effects for both drugs. The within-subject crossover design where participants serve as their own control will greatly reduce variability due to individual differences in drug metabolism, sensitivity, and other factors, and increase statistical power. The cannabis doses were informed based on our prior research (Vandrey et al., 2017; Spindle et al., 2018; Spindle et al., 2021), which showed that 10 mg Δ9-THC elicits discriminable drug effects but little to no impairment while 25 mg Δ9-THC reliably impairs cognitive/psychomotor performance. Thus, these two doses are ideal for testing the sensitivity of the various performance measures in this study. Moreover, these Δ9-THC doses are ecologically valid, as they are common among retail cannabis products sold in dispensaries (Hammond, 2021). Target BACs were selected based on *per se* limits for alcohol in most U.S. states (0.08%) and most of Europe (0.05%). Dosing participants to a 0.05% BAC in alcohol and cannabis co-use conditions will also avoid ceiling effects on study outcomes and mitigate potential untoward drug effects (e.g., emesis) that may be present if higher alcohol doses were given with cannabis. This study design will also reveal whether alcohol (at a BAC below the U.S. legal limit for DUI) and oral/vaporized cannabis have additive or synergistic effects when combined that meet or exceed the effects produced by alcohol alone at a higher BAC (0.08%) that does constitute DUI. Lastly, the proposed timing of cannabis and alcohol administration will ensure that peak cannabis effects (for both oral and vaporized cannabis) coincide with peak alcohol effects, thus capturing the “worst-case scenario” in terms of impairment from alcohol/cannabis co-use for both routes of administration.

The proposed study population was also carefully considered. The cannabis-use criteria of study participants (i.e., ≤ 2 uses/week but ≥ 5 uses in the past year) will constrain the impact of Δ9-THC tolerance on behavioral outcomes while ensuring that participants are not inexperienced with cannabis. Participants will be required to report two recent binge drinking episodes and to have co-used alcohol and cannabis at least once in the past year; these criteria will further increase the external validity of this study, as binge drinkers and co-users are highly likely to report driving under the influence of cannabis and/or alcohol (Metrik et al., 2018; Patrick et al., 2018). The binge drinking criteria will also ensure that study participants can tolerate the two alcohol doses. Non-drinkers and people under the age of 21 will not be included for ethical and legal reasons. Those meeting criteria for severe AUD will not be eligible to limit potential study confounds (e.g., these individuals would likely have greater alcohol tolerance and complications with withdrawal during abstinence). Overall, the study population is ideal for: safely administering the proposed alcohol and cannabis doses and reducing the likelihood of adverse effects or untoward drug effects, maximizing our ability to detect impairment, and increasing generalizability of our findings to individuals most likely to co-use these drugs and drive while impaired.

A key reason we decided to write this protocol paper was to use this as a platform to describe the custom driving scenarios we created for this project and to share the drives with researchers interested in using them. Though there have been many well-designed and informative prior studies on simulated driving impairment from alcohol, cannabis, or other drugs, there has historically been little standardization across experiments and laboratories with respect to the types of driving simulations tested. The considerable heterogeneity of driving simulations used in prior research often makes it difficult to compare outcome measures across studies. Beyond having few publicly available driving simulation examples, another notable hindrance to conducting driving simulator research is the lack of tools to facilitate data extraction and organization. For example, the STISIM software collects data every 0.1 s (10 Hz), generating thousands of lines of data for each completed drive. Oftentimes there is a need to examine a particular outcome measure at a specific driving segment (e.g., SDLP during a divided attention task, reaction time to a stimulus), which requires the creation of complex programs to extract, calculate, and organize the data appropriately; few, if any, of these programs are publicly available to researchers. For this project, using the STISIM coding language, we created 10 unique, yet comparable, driving simulations (each about 25 min in duration) that test key aspects of performance shown to be impacted by cannabis and alcohol. We have shared these drives (see Methods) along with a custom an R script that can extract and organize the data into an excel spreadsheet. These simulations require participants to navigate various environments (e.g., residential, rural, and metro areas) where they interact with signal lights, stop signs, and respond to unexpected events (e.g., pedestrians crossing the street); data is collected at specific events of interest that may be impacted by alcohol/cannabis intoxication. These drives also include two controlled driving scenarios (car following task and divided attention task) demonstrated to be sensitive to alcohol/cannabis effects. The 10 versions of the driving simulation were created to mitigate practice/learning effects and differ only by the order that events/scenarios occur and by the type of accident avoidances the driver must respond to. We encourage any researchers interested in using these drives to contact us in the interest of promoting cross-study comparisons and future collaborations.

There are several alternate design options beyond the scope of this project that may be worth exploring in future studies. For example, this study will only administer cannabis that contains high Δ9-THC concentrations and low concentrations of CBD and other cannabinoids. Additional studies are needed to understand how other cannabis chemotypes (e.g., high CBD/low Δ9-THC) interact with alcohol. Moreover, additional studies are needed to understand how cannabis impairment differs in older populations (over 55 years old) or those with more frequent (e.g., daily) cannabis use patterns than the target sample for this research. Lastly, though the use of a driving simulator is safe and provides excellent experimental control, it likely cannot fully capture the complexity of actual driving. On-road studies examining the effects of alcohol and oral/vaporized cannabis will be an important follow-up to this research.

Overall, this project can improve public safety, including by informing various regulatory decisions regarding cannabis. First, this project can inform impairment detection standards for alcohol and cannabis. For example, should alcohol/cannabis co-use (at a 0.05% BAC) produce additive or synergistic impairment that is commensurate with impairment from alcohol alone at a 0.08% BAC (the current *per se* limit for impairment in the United States), these results would suggest a lower *per se* limit may be needed for those who have co-used alcohol and cannabis. In addition, this project will further evaluate a novel cannabis impairment detection tool (the DRUID) that has shown promise in preliminary research (Spindle et al., 2021). Further enhancement of this instrument could improve public safety because there are currently no empirically validated methods for identifying impairment from cannabis. Second, results can assist regulators faced with future decisions regarding the allowance of commercial establishments that sell alcohol and cannabis or products that contain both alcohol and Δ9-THC. Finally, this study will provide key insights that will be critical to relay to the general public such as: 1) differences in the magnitude, onset, and duration of driving impairment from oral and vaporized high Δ9-THC cannabis, 2) the extent to which cannabis impairment is augmented when co-used with alcohol, and 3) the relation between one’s perceived level of cannabis impairment versus their objective level of impairment. Ultimately, given the unprecedented and growing number of people with legal access to cannabis, communicating information related to cannabis-induced impairment *via* mass media campaigns or other accessible channels will be critical to mitigating cannabis-related harms.
